# Low-Cost Spray-Patterned Triboelectric Textiles for Wearable Interaction and Energy Harvesting

**DOI:** 10.3390/s26175554

**Published:** 2026-09-01

**Authors:** Hebo Gong, Shijian Luo, Ping Shan

**Affiliations:** College of Computer Science and Technology, Zhejiang University, Hangzhou 310027‌, China; gonghebo8@zju.edu.cn (H.G.); shanping@zju.edu.cn (P.S.)

**Keywords:** triboelectric nanogenerator, textile sensor, spray coating, wearable interaction, gesture recognition, energy harvesting, self-powered sensor, rapid prototyping

## Abstract

Smart textile interfaces hold promise for battery-free wearable interaction, yet their adoption is limited by complex fabrication and insufficient on-body evaluation. We present TriboTex, a low-cost spray-patterning workflow that forms nylon–Cu–nylon triboelectric stacks on cotton textiles using laser-cut PET stencils and commercially available materials. The core consumables cost approximately USD 0.003/cm^2^, and sensor geometry can be rapidly iterated by modifying only the digital stencil. Controlled characterization across nine devices from three fabrication batches showed a peak open-circuit voltage of 52.3 V and a maximum power density of 1870 µW/m^2^ at 4 GΩ. The output retained 96.1% of its initial voltage after 1000 bending cycles and 94.2% after 24 h of simplified saline immersion. Three-sample environmental sweeps showed voltage amplitudes of 41.9–43.7 V from 15 to 45 °C, with a decrease to 27.7 V at 0 °C; the humidity response remained within 92.7–104.5% of the 20% RH value over 20–60% RH but decreased to 19.9% at 70% RH. Two wearable prototypes were developed: a single-electrode garment sleeve recognized tap, double-tap, and swipe gestures with 95.0% accuracy across 1200 trials from 12 participants; a single-electrode insole generated action-dependent peak voltages up to 123 V under repeated foot loading and was connected through a rectification and voltage-regulation module to charge a battery. Across the two 12-participant studies, attachment and fit stability emerged as shared integration requirements, while participant feedback and controlled humidity measurements highlighted moisture management as a priority for reliable on-body sensing and energy capture. The primary contribution is an accessible, low-cost, and geometry-flexible route for early-stage wearable sensing experiments and application demonstrations, supported by documented fabrication, electrical characterization, and human-centered evaluation.

## 1. Introduction

Wearable textiles can distribute sensing over garments and accessories while remaining conformal to the body. Triboelectric devices are particularly relevant to this design space because contact electrification and electrostatic induction convert touch, pressure, and motion into electrical signals without a powered sensing element at the point of contact [1,2]. Textile TENGs have consequently been studied for touch and gesture input [3,4,5], gait sensing [6], and biomechanical energy capture [7,8].

Fabrication remains a practical barrier. Printing, lamination, weaving, embroidery, transfer, and coating can produce functional textile TENGs, but each route imposes material, equipment, registration, curing, or bonding requirements [9,10]. Wearable interface research also requires frequent changes to sensor shape and body placement. A fabrication process that retains the same deposition sequence while changing the patterned geometry can support this iterative design process. Screen-printed, digitally printed, and sprayed textile devices illustrate how patterned deposition can support this design space [11,12,13].

Electrical characterization and wearable validation address complementary questions. Open-circuit voltage, short-circuit current, transferred charge, load matching, durability, and wash response describe device behavior under controlled conditions. On-body studies reveal how material stiffness, attachment, fit, sweat, and movement affect an integrated interface. The garment and insole both use single-electrode sensing but experience different mechanical inputs: direct touch and sliding on the garment and repeated vertical contact–separation loading in the insole. The insole output is subsequently rectified and voltage-regulated for battery charging.

**Figure 1:**
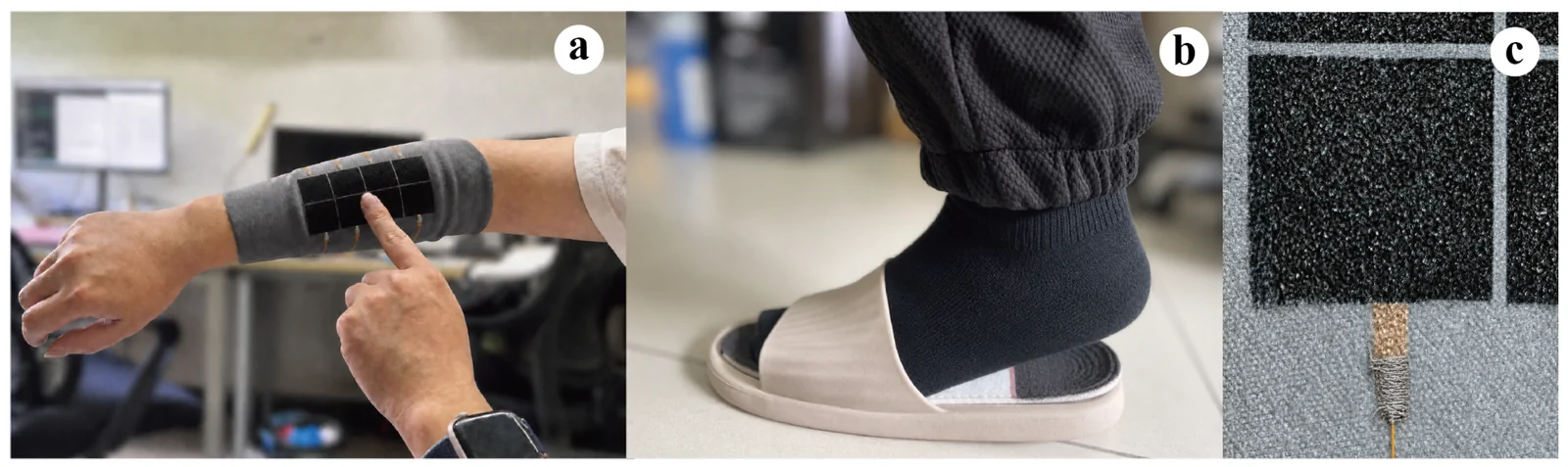
TriboTex wearable prototypes. (**a**) Eight-region single-electrode sleeve for tap, double-tap, and swipe input. (**b**) Single-electrode insole integrated into the test footwear and mechanically excited by repeated vertical contact–separation loading. (**c**) Detail of a patterned textile region and conductive connection.

We developed TriboTex to connect these fabrication, electrical, and wearable requirements. The process uses an adhesive PET stencil to spray-pattern a nylon–Cu–nylon functional stack on cotton cloth. Designers can alter the active geometry by editing and recutting the stencil while retaining the same spray and curing sequence. Figure 1 shows the two application prototypes evaluated in this study.

The electrical measurements establish the functionality and operating range of the resulting devices. TriboTex contributes a combination of commercially accessible materials, low equipment requirements, documented consumable cost, and rapid stencil-based geometry revision for wearable demos and early-stage experimental iteration.

This paper makes three contributions:

1. A validated stencil-guided spray-patterning workflow for rapid, geometry-flexible triboelectric-textile prototyping, with commercially accessible materials, recorded fabrication parameters, and a consumable cost of USD 0.003/cm^2^. Sensor geometry can be revised through the digital stencil without changing the material preparation, layer sequence, or curing procedure.
2. A quantitative fabrication and reproducibility characterization that relates nylon concentration to layer formation and pattern area and shape to electrical output, and demonstrates low cycle-to-cycle, device-to-device, and batch-to-batch variation under controlled excitation across nine devices from three independently fabricated batches.
3. Wearable-integration insights from two single-electrode prototypes and two 12-participant studies: attachment and fit stability emerged as a shared requirement that manifests differently by mechanical mode—functional degradation in the garment, comfort degradation in the insole—while controlled humidity characterization explains perspiration-related recognition errors reported in the garment study, identifying moisture management as an integration priority not yet evaluated for the insole.

## 2. Related Work

### 2.1. Textile Triboelectric Sensors and Generators

TENGs convert mechanical contact, separation, sliding, or deformation into electrical output through coupled contact electrification and electrostatic induction. The theoretical conversion process has been formalized through contact-mode TENG models [14]. When two materials with different electron affinities make contact, charge transfers across their interface. Their subsequent separation changes the electric potential and drives current through the external circuit [15]. The polarity and magnitude of the response depend on the material pair, effective contact area, surface condition, mechanical input, electrode configuration, and connected electrical load. This combination of mechanical and electrical factors makes operating-mode selection central to the design of a textile TENG [16].

The four common operating modes are vertical contact–separation, lateral sliding, single electrode, and freestanding triboelectric layer [17]. Single-electrode structures connect one electrode to the measurement circuit while the contacting body or object acts as the moving triboelectric counterpart. This arrangement reduces wiring at the sensing surface and suits distributed touch regions on garments [10,18]. Vertical contact–separation structures use two opposing triboelectric layers whose periodic compression and release generate alternating output. Their motion corresponds directly to repeated plantar loading in footwear [6,7]. TriboTex uses single-electrode mode for both the garment and the insole. In the insole, vertical contact–separation describes the mechanical excitation during plantar loading, not a two-electrode circuit configuration.

Textile implementations extend these mechanisms to flexible substrates, functional fibers, yarns, and surface coatings. Reported systems use material pairs including nylon, PTFE, silicone, conductive yarns, and carbon- or graphene-based coatings [13,19,20,21]. Textile substrates also introduce characteristics that are less prominent in rigid devices: yarn-scale roughness changes the real contact area, fabric deformation alters the separation trajectory, and the coating or electrode can change drape, thickness, breathability, and skin feel [9,22,23]. A fabrication method must therefore form the electrical stack while preserving enough mechanical compliance for the intended body location.

Wearable TENGs have detected walking and gait events and captured mechanical energy from repeated body motion [6,21,24]. In interactive garments, an isolated voltage peak can encode a touch event, while multiple spatially distributed electrodes provide channel identity, peak timing, and activation order. These features support gesture vocabularies on sleeves, gloves, and other textile surfaces [3,5,25,26]. Footwear systems experience larger compressive inputs and can combine event detection with rectified energy storage. The sensor and energy-capture functions share the triboelectric mechanism but impose different requirements on electrode layout, wiring, mechanical packaging, and downstream electronics.

Within these interactive applications, TENG recognition systems range from deterministic event logic to learned classifiers. Simple interfaces commonly detect peaks and then use amplitude, duration, inter-peak interval, or channel activation order as decision variables. This approach is computationally inexpensive and interpretable, although fixed thresholds can be sensitive to user, force, humidity, and placement variation. Traditional machine-learning pipelines instead extract temporal or spectral features before classification; examples include wavelet features with ensemble learning for collision-target classification and a machine-learning-assisted wristband for full-keyboard and multicommand gesture input [27,28]. Deep-learning methods have further been applied to multichannel rescue-gesture sensing, combined contact–sliding signals for material and texture identification, non-driving hand-behavior recognition, and gesture biometric authentication using a lightweight network [29,30,31,32]. At the system level, recent reviews extend this progression toward AI-enabled self-powered sensing and wearable edge intelligence, emphasizing the joint design of sensor signals, preprocessing, model complexity, and power budget [33,34].

Here, the fixed-rule recognizer serves as an evaluation instrument for the patterned textile. It uses peak count, inter-peak timing, and channel activation order to determine whether the spray-patterned regions produce distinguishable signals for a three-gesture demonstrator. Adaptive thresholds or lightweight learned models could be considered when extending the system to larger gesture vocabularies and less constrained garment placement.

### 2.2. Textile TENG Fabrication Methods

Fabrication routes determine pattern resolution, equipment requirements, material compatibility, textile hand, and the effort required to revise a layout. Section 5.1 provides a structured comparison; here, we summarize each category to establish context.

Screen and digital printing deposit functional inks through a patterned mesh or inkjet printhead, followed by curing. Screen printing offers repeatable deposition but requires a new screen for each geometry; digital printing edits the pattern through the print file at the cost of stricter ink–printhead compatibility [3,11,12].

Electrospinning draws polymer solutions through a high electric field to form micro- or nanofibrous mats with high surface area. Patterning requires shaped collectors, masks, or post-cutting and transfer [9,35].

Dip-coating immerses yarn or fabric in a functional solution, providing conformal coverage with compact equipment. Selective sensing regions require masking or post-patterning [36,37].

Weaving, knitting, and embroidery integrate functional yarns directly into the textile structure through programmed interlacing, loop formation, or stitching. Geometry changes require revised machine programs and yarn setups [9,38,39].

Lamination and transfer assemble preformed conductive and triboelectric layers through cutting, alignment, and bonding. Geometry revision requires recutting and realigning component layers [10].

Spray coating atomizes a liquid material onto a flexible surface through masks or stencils. A digitally cut stencil exposes sensor geometry as an editable input while retaining the same coating equipment and layer sequence [13,40,41].

These routes differ in where process repeatability is established and when sensing regions enter the textile-production sequence. Post-fabrication deposition methods (printing, spraying, transfer, lamination) can functionalize an existing fabric, supporting placement decisions after a garment layout has been selected. However, the added layers alter local mechanical properties, linking the fabrication route directly to wearability.

Recent HCI research has examined accessible tools for designing triboelectric interactions and textile sensing systems [42,43]. TriboTex follows this direction with adhesive PET stencils and sequential spray deposition: the digital stencil changes while the nylon–Cu–nylon materials, spray settings, and curing sequence remain fixed.

## 3. Materials and Methods

### 3.1. Fabrication Workflow

**Figure 2:**
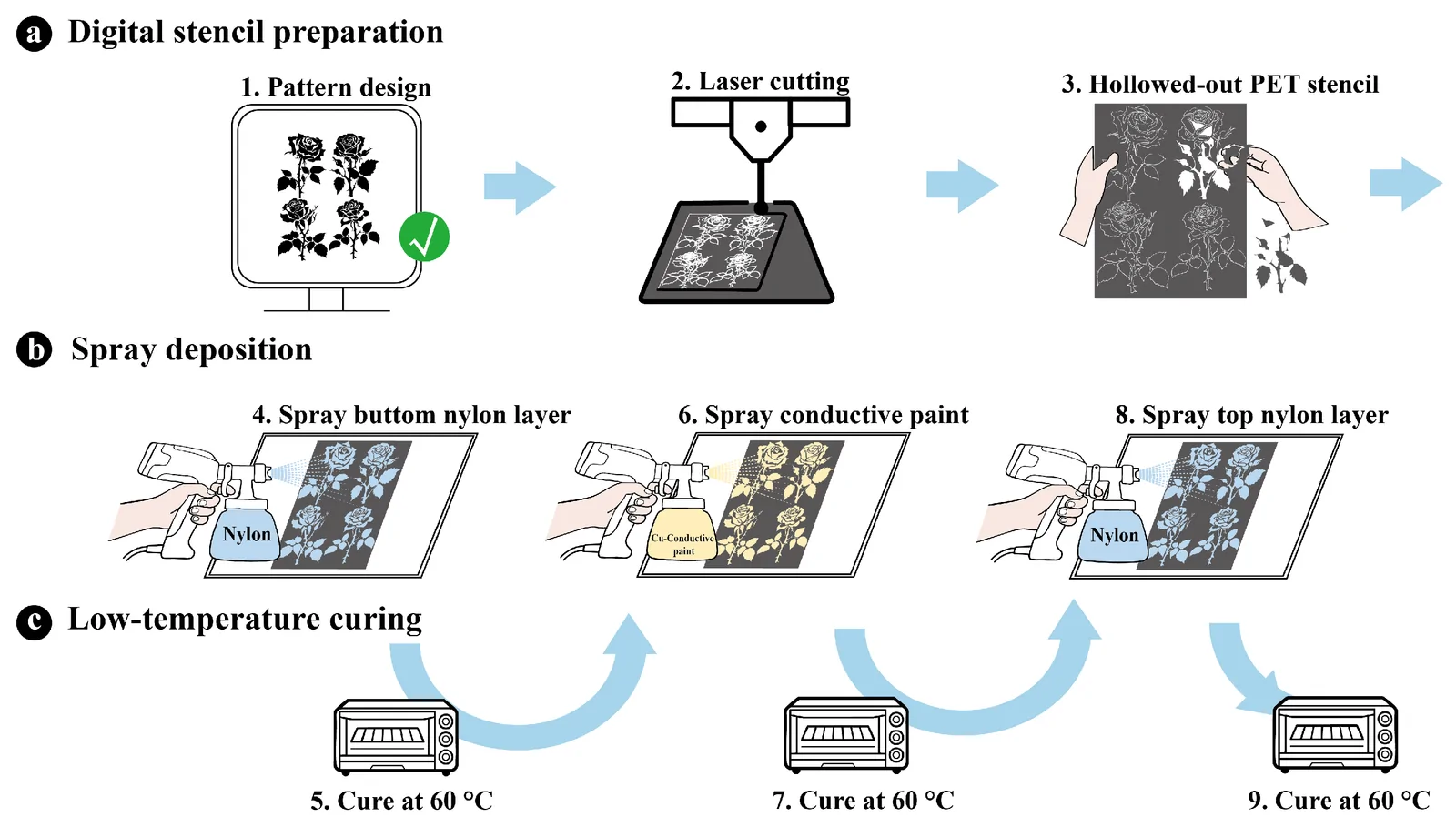
Stencil-guided TriboTex fabrication workflow: digital pattern preparation, adhesive PET stencil cutting and alignment, sequential nylon, Cu-based, and nylon spray deposition, and low-temperature curing. Arrows indicate the process sequence, and the green check mark indicates completion of the digital pattern before laser cutting.

TriboTex uses a cotton substrate and a nylon–Cu–nylon functional stack. The bottom nylon layer separates the conductive coating from the textile substrate, the middle Cu-based coating collects the induced signal, and the top nylon layer forms the primary triboelectric contact surface. Figure 2 summarizes the fabrication sequence.

The workflow began with a vector pattern defining the active region and electrical connection path. The pattern was laser-cut from adhesive PET, aligned with the cotton substrate, and retained throughout sequential deposition of the bottom nylon layer, Cu-based electrode, and top nylon layer. Supplementary Table S1 provides the stencil dimensions used for the prototypes, and Table 1 summarizes the detailed fabrication parameters. Revising the sensor geometry requires only modification of the vector pattern and PET stencil, while the material preparation, deposition sequence, and curing procedure remain unchanged. For the standard 3 cm × 3 cm specimens used in electrical characterization, the complete workflow was finished in less than 120 min, including stencil cutting and alignment, sequential spraying, and three 20-min layer-drying steps.

The settings in Table 1 were established as a reproducible working protocol through preliminary fabrication trials rather than a full factorial optimization. Preliminary drying trials were conducted at four temperature conditions before the standard protocol was established. At 50 °C, a 20-min cure did not fully dry the coating. At 70 °C, 15-min curing produced a visibly stiffened textile with a harder handle. At 80 °C, 15-min curing produced visible scorching of the cotton substrate. At 60 °C, a 20-min cure achieved complete drying without visible thermal damage. Based on these trials, 60 °C for 20 min was adopted as the standard curing condition for all layers (Supplementary Table S13). These observations were recorded qualitatively by visual and tactile assessment rather than by instrumented color or mechanical measurement. The selected condition therefore represents an operational setting validated for the present textile, coating, and oven configuration rather than a globally optimized parameter.

Before device fabrication, we screened nylon solutions at 70%, 60%, 50%, and 40% concentration. We deposited each screening coating on a glass slide and dried it at 60 °C for 15 min; this screening condition was separate from the 20-min interlayer cures used for complete devices. For each concentration condition, thickness was measured at five locations on the corresponding coating. The average measured thicknesses were 64.3, 59.2, 57.6, and 51.2 µm, respectively. The 40% and 50% solutions formed thinner coatings but produced incomplete coverage when applied over the Cu-based layer. The 70% solution formed continuous coverage with the greatest measured thickness. We selected the 60% solution because it maintained continuous coverage with a thinner coating than the 70% condition. Thus, nylon concentration was the fabrication variable quantitatively screened in this study. Full thickness measurements and the shape-by-size screening results are provided in Supplementary Figure S3. As with the curing temperature, the laser-cutting parameters (power, speed, and pass count) and spray-deposition parameters (pass count, distance, and angle) reported in Table 1 were established through directed preliminary trials targeting complete stencil cutting and continuous coating without visible defects, rather than through systematic parameter sweeps. Only the adopted settings were retained as part of the standard protocol. A full factorial optimization of these parameters is identified as future work in Section 5.2.

### 3.2. Materials and Equipment

The fabrication materials comprised commercial cotton textile, MIHE waterproof nylon ink (Hangzhou Mihe Trading Co., Ltd., Hangzhou, China), RoHS-compliant 718 medium-drying diluent (Tuye Chemicals (Guangzhou) Co., Ltd., Guangzhou, China), Cu-based conductive aerosol coating (Shenzhen Jingzhe Technology Co., Ltd., Shenzhen, China), and 0.225-mm adhesive PET stencil film (Darit Tape Adhesive Products Co., Ltd., Shenzhen, China). The reusable fabrication equipment comprised an xTool D1 Pro laser cutter (Makeblock Co., Ltd., Shenzhen, China), an electric spray gun (Delixi Electric Co., Ltd., Wenzhou, China), and a drying oven operated at 60 °C. The cotton textile and drying oven were existing laboratory resources for which supplier records were not retained. The documented material cost was USD 0.003/cm^2^ (consumables only); Supplementary Table S2 gives the purchase-condition calculation and excluded items.

For bench characterization, we used a PTFE film as the counter-contact, a TN16×10S pneumatic cylinder (16-mm bore, 10-mm stroke; Zhejiang Bobo Pneumatic Technology Co., Ltd., Wenzhou, China), an oil-free air compressor (Taizhou Outstanding Industry & Trade Co., Ltd., Taizhou, China), a solenoid valve, and an HZC-H1-10KG load cell (Bengbu Chengying Sensor Co., Ltd., Bengbu, China; 0–10 kg range). A Keithley 6517B electrometer (Keithley Instruments, Inc., Cleveland, OH, USA) measured open-circuit voltage (*V*_OC_), short-circuit current (*I*_SC_), and transferred charge (*Q*_SC_).

### 3.3. Electrical Characterization Setup

The tested sample contained a 30 × 30 mm active region on a 40 × 40 mm textile substrate. A 0.08-mm-thick PTFE counter-contact measured 42 × 17 mm and overlapped the TriboTex surface over 30 × 17 mm (5.10 cm^2^). All controlled electrical measurements used a single-electrode configuration: the Cu electrode in TriboTex was connected to the electrometer, while the PTFE film served as the electrically unconnected moving triboelectric counter-contact. The pneumatic cylinder generated periodic vertical contact and separation over a nominal distance of 10 mm, and the load cell verified the force. Supplementary Figure S2 shows the complete apparatus.

For the force sweep, we applied 10–50 N in increments of 10 N at 1 Hz. For the frequency sweep, we applied 1–4 Hz in increments of 1 Hz at 20 N. Unless stated otherwise, the laboratory conditions were approximately 25 °C and 40% relative humidity. The archived load sweep contains 33 external-resistance points from 1 kΩ to 70 GΩ. We calculated device current, load power, and area-normalized power density as

(1) $$I=JA,\;P=I^{2}R,\;p_{A}=\frac{P}{A},$$

where *J* is current density, *A* = 5.10 × 10^−4^ m^2^, and *R* is load resistance.

**Figure 3:**
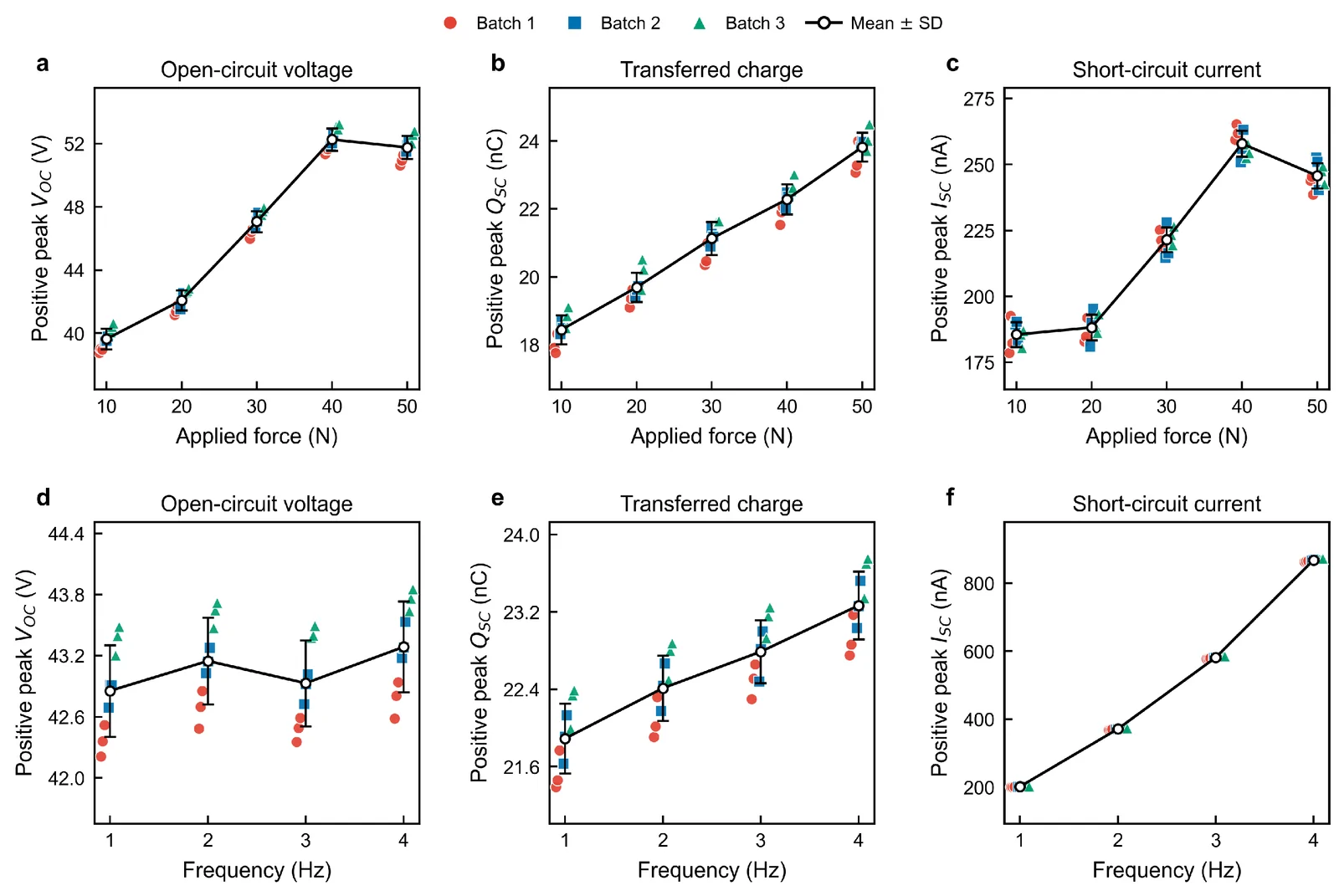
Electrical output and device-to-device repeatability under controlled excitation. (**a**–**c**) Positive peak *V*_OC_, *Q*_SC_, and *I*_SC_ at 10–50 N and 1 Hz. (**d**–**f**) Corresponding positive peaks at 1–4 Hz and 20 N. Symbols identify nine devices from three fabrication batches; black circles and error bars show the mean ± sample standard deviation across the nine devices.

For the force- and frequency-response repeatability analysis, nine devices were prepared in three fabrication batches, with three devices per batch. For each device and condition, positive peaks were extracted from the recorded voltage, transferred-charge, and current data. Figure 3 reports the individual device values together with the mean and sample standard deviation across the nine devices. Peak-amplitude repeatability was quantified as the coefficient of variation, $\mathrm{CV}=100s/\bar{x}$. Within-device CV was calculated from repeated positive peaks for each device and condition and then summarized across the nine device-level CVs. Inter-device CV was calculated from the nine device-level means, whereas batch-to-batch CV was calculated from three batch means, each based on three devices. Sample standard deviation was used throughout; the three-batch CV is treated as a descriptive measure. Full results are provided in Supplementary Table S12.

Unless otherwise stated, electrical measurements for the wash, bending-cycle, and saline-immersion tests were performed under a 20 N contact force at 1 Hz.

For wash testing, we hand-washed the sample for 10 min in water containing detergent and dried it naturally before each measurement. We recorded the original response, the response after one cycle, and the response after five cycles. For mechanical cycling, we actuated the sample for 8000 contact–separation cycles at 2 Hz and summarized each consecutive block of 1000 measurements.

For bending durability, three TriboTex samples were manually bent around a 10-mm-diameter metal rod at approximately 1 Hz. Bending and subsequent return to the flat state were counted as one cycle. Each sample underwent 1000 cycles, with voltage and current recorded at 0, 200, 600, and 1000 cycles. Ten readings were obtained from each sample at every checkpoint. The plotted means and standard deviations were calculated from the 30 readings at each checkpoint. Static bending-angle resistance was additionally recorded at 0°, 30°, 60°, and 90°, with five repeated readings at each angle. The resistance change was normalized to the mean value at 0°.

For the saline-immersion test, a 1 wt% NaCl solution was prepared using salt and purified water. Twelve samples were assigned to 1, 4, 8, and 24 h static immersion at approximately 37 °C, with three samples per exposure duration and a sample-to-liquid bath ratio of 1:50. After immersion, each sample was gently rinsed with deionized water for 10 s, dried at 40 °C for 30 min, and electrically retested. For each condition, 18 voltage peaks were identified in each of three recorded traces. The plotted mean and standard deviation were calculated from the 54 detected peaks.

For humidity-response testing, the mechanical loading apparatus was placed in a sealed plastic enclosure. Relative humidity (RH) was first reduced to approximately 20% using a DH-CS02 dehumidifier (Guangdong DOUHE Technology Co., Ltd., Foshan, China) and was then increased stepwise using a Mijia Humidifier 2 (MJJSQ06DY, Xiaomi Corporation, Beijing, China). RH was monitored using a Mijia Bluetooth Temperature and Humidity Monitor 2 (Xiaomi Corporation, Beijing, China). Voltage waveforms were recorded after each target level of 20%, 30%, 40%, 50%, 60%, and 70% RH was reached, while the mechanical loading settings were held at 20 N and 1 Hz. For each of the three samples, the voltage amplitude of each valid cycle was calculated as the local peak minus the baseline defined by the adjacent troughs. Cycle amplitudes were averaged within each sample, after which the mean and sample standard deviation were calculated across the three samples.

For temperature-response testing, three TriboTex samples were placed on a cooling/heating platform that locally controlled the textile region under test. Voltage waveforms were recorded at target temperatures of 0, 15, 30, and 45 °C under 20 N and 1 Hz mechanical loading. The voltage amplitude was extracted using the same local peak-to-adjacent-trough procedure and was first averaged within each sample; the reported mean and sample standard deviation were then calculated across the three sample-level values. This configuration provided local thermal exposure rather than whole-enclosure temperature conditioning.

### 3.4. Wearable Prototype Design

The garment prototype used eight spray-patterned regions in single-electrode mode. Two identical four-channel analog front ends conditioned the high-impedance signals, and a Seeed Studio XIAO microcontroller (Seeed Technology Co., Ltd., Shenzhen, China) sampled each channel at 60 Hz (480 samples/s aggregate) and streamed the data to a host computer. After conversion to the sensor-voltage scale, the computer used fixed thresholds of 30 V for tap and double-tap and 25 V for swipe. A localized activation above 30 V was classified as a tap. Two such peaks within the same recognition window were classified as a double-tap only when they were separated by a distinct trough that returned sufficiently close to the baseline; a swipe was identified when three sensing regions in the same column exceeded 25 V in spatial sequence. These deterministic threshold and sequence rules provided transparent application-level validation of the patterned sensing layout. Supplementary Figure S4 presents recorded multichannel waveform examples, and Supplementary Table S3 reports the analog front-end and acquisition parameters.

The insole prototype also used single-electrode mode and was mechanically excited by repeated vertical contact and separation during foot loading. Its alternating open-circuit voltage was measured directly, or its output was passed through a rectification and voltage-regulation PCB connected directly to a battery. Supplementary Figure S5 shows the garment electronics, while Supplementary Figure S6 shows the insole charging circuit and battery connection.

### 3.5. User Study Protocol

Twelve adults completed the garment study (age 29.0 ± 5.2 years; six male and six female; ten right-handed and two left-handed). Each participant performed 100 trials after learning the three gestures. The complete dataset contained 384 taps, 405 double-taps, and 411 swipes. We compared the predicted and intended labels and then collected a 7-point Likert questionnaire and open-ended feedback.

A separate group of 12 adults completed the insole study (age 32.3 ± 10.0 years; six female and six male; daily shoe size 40.4 ± 2.9, range 36–45). Participants wore the insole for 10 min and walked indoors; four also used stairs, and one also ran or jumped. The test used open-toe slippers in sizes 38 and 43. Participants then completed a 7-point Likert questionnaire and open-ended feedback.

All participants provided written informed consent. We collected no personally identifiable information and anonymized the study records before analysis. Participant-level recognition data, questionnaire items, and complete response matrices are provided in Supplementary Tables S4–S10.

## 4. Results

### 4.1. Electrical Performance

Figure 3 summarizes the force- and frequency-response measurements for nine devices from three fabrication batches. At 1 Hz, mean *V*_OC_ increased from 39.63 ± 0.65 V at 10 N to 52.26 ± 0.71 V at 40 N and measured 51.76 ± 0.73 V at 50 N. Mean *Q*_SC_ increased from 18.44 ± 0.43 nC to 23.82 ± 0.42 nC across 10–50 N. Mean positive *I*_SC_ increased from 185.50 ± 4.75 nA at 10 N to 257.92 ± 4.92 nA at 40 N and measured 245.71 ± 4.82 nA at 50 N.

At 20 N, mean *V*_OC_ remained between 42.85 and 43.29 V over 1–4 Hz, while mean *Q*_SC_ increased from 21.89 ± 0.36 nC to 23.27 ± 0.35 nC. Mean positive *I*_SC_ increased from 202.14 ± 0.63 nA to 867.65 ± 3.34 nA, consistent with faster charge transfer at higher actuation frequency. Representative time-domain waveforms are provided in Supplementary Figure S7.

The hierarchical CV analysis quantified peak-amplitude consistency under the same controlled excitation. Across the 10–50 N force sweep at 1 Hz, the mean within-device CV for positive current peaks was 0.18–0.25%, the inter-device CV was 1.91–2.58%, and the descriptive batch-to-batch CV was 0.75–1.47%. Across 1–4 Hz at 20 N, the corresponding ranges were 0.05–0.21%, 0.31–0.43%, and 0.34–0.47% for current, and 0.98–1.08%, 0.98–1.05%, and 1.08–1.17% for voltage. These values indicate limited cycle-to-cycle, device-to-device, and batch-to-batch dispersion under the tested bench conditions (Supplementary Table S12).

**Figure 4:**
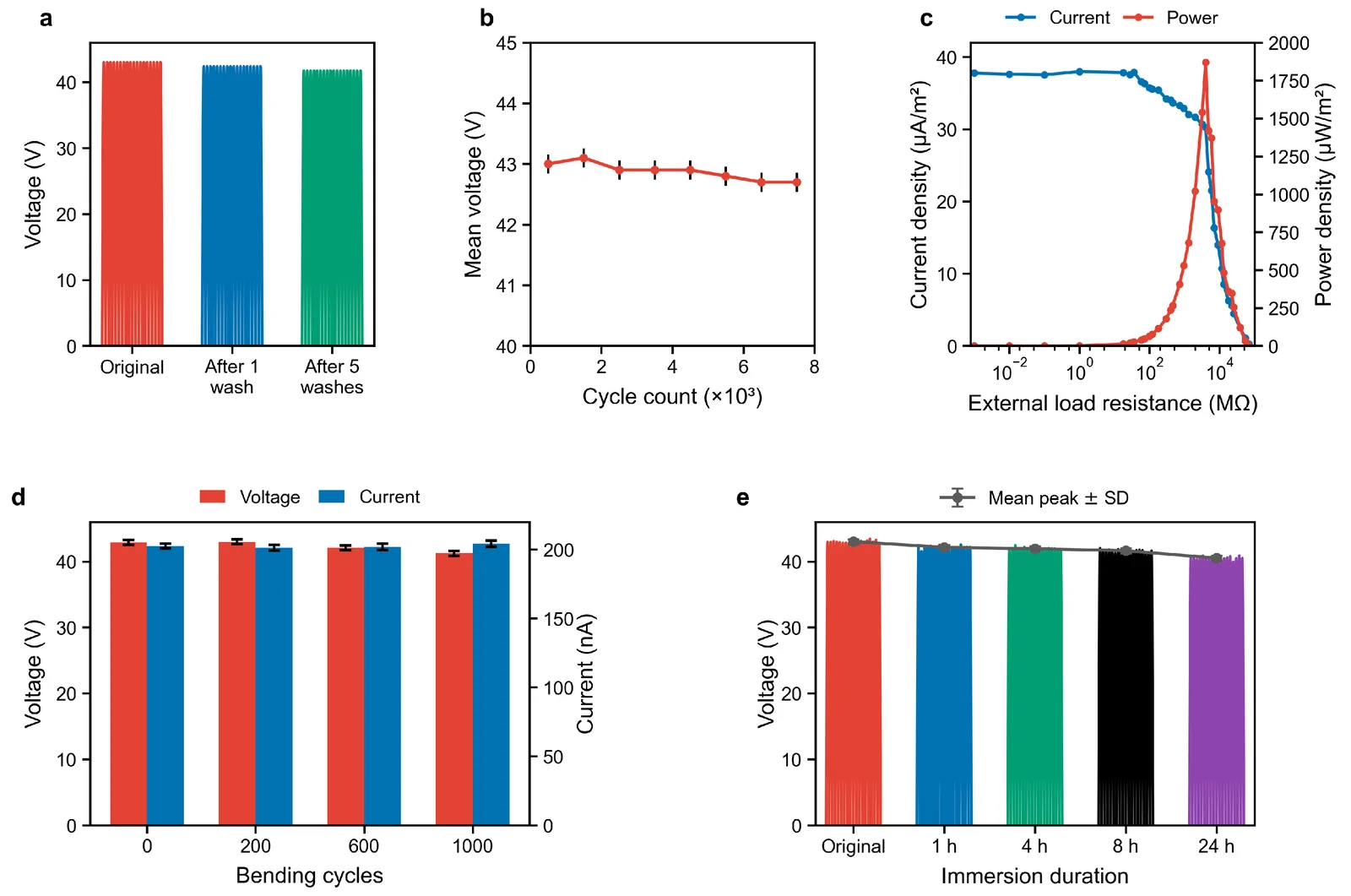
Durability and load-dependent output. Wash, bending-cycle, and saline-immersion measurements used 20 N and 1 Hz. (**a**) Voltage before washing, after one hand-washing cycle, and after five cycles; each cycle comprised 10 min of hand washing in detergent solution followed by natural drying. (**b**) Mean voltage over 8000 contact–separation cycles at 2 Hz; each point summarizes 1000 measurements, and error bars show one sample standard deviation within that interval. (**c**) Current density and power density from the 33-point load sweep over 1 kΩ–70 GΩ. (**d**) Voltage and current measured at 0, 200, 600, and 1000 manual bending cycles; error bars show the sample standard deviation across 30 readings from three samples. (**e**) Voltage waveforms and mean peak voltage after 0, 1, 4, 8, and 24 h of static immersion in 1 wt% NaCl; error bars show the sample standard deviation across 54 detected peaks from three traces per condition.

Figure 4c shows the external-load sweep from 1 kΩ to 70 GΩ. The maximum power density was 1870 µW/m^2^ at 4 GΩ. At this load, the measured current was 15.44 nA and the calculated load voltage was 61.76 V, corresponding to an absolute output power of 0.954 µW for the tested 5.10 cm^2^ contact area. Thus, 4 GΩ was identified as the load-matching condition for the tested device and mechanical excitation.

The pattern-screening measurements confirmed that the spray-patterning process produced consistent electrical responses across different geometries. Using the selected 60% nylon solution, we fabricated square, circular, and triangular patterns at matched nominal areas of 4, 9, 16, and 25 cm^2^. Five open-circuit-voltage measurements were averaged for each condition. For square patterns, the mean *V*_OC_ increased monotonically from 17.3 V at 4 cm^2^ to 80.0 V at 25 cm^2^. At the largest nominal area, the circular pattern produced 83.9 V and the triangular pattern produced 74.6 V. All three shapes showed increasing voltage with nominal area. At matched nominal areas, the circular patterns produced the highest values, followed by the square and triangular patterns. The complete shape-and-area series is presented in Supplementary Figure S3.

### 4.2. Durability and Environmental Response

Figure 4a shows peak voltages of 43.00 V before washing, 42.37 V after one hand-washing cycle, and 41.74 V after five cycles. The five-cycle retention was approximately 97.1%. During the 8000-cycle test at 2 Hz, the mean voltage changed from 43.0 V in the first 1000 cycles to 42.7 V in the final 1000 cycles, corresponding to approximately 99.3% retention (Figure 4b). Error bars show one sample standard deviation within each 1000-measurement block.

Figure 4d presents the additional bending-cycle test. The mean voltage changed from 42.89 ± 0.37 V before bending to 41.23 ± 0.37 V after 1000 cycles, corresponding to 96.1% retention. The mean current changed from 202.50 ± 1.84 nA to 204.23 ± 2.19 nA over the same test. The slight increase in current was within the measurement uncertainty and was not interpreted as a physically meaningful change. Under static bending, the normalized resistance change increased from 2.68% at 30° to 8.04% at 60° and 9.82% at 90° (Supplementary Figure S8). Figure 4e shows the saline-immersion response. Mean peak voltage decreased gradually from 43.00 ± 0.37 V for the original condition to 40.51 ± 0.37 V after 24 h, corresponding to 94.2% retention. Supplementary Figure S9 summarizes the humidity and local-temperature responses across three samples. The mean baseline-corrected voltage amplitude was 36.01 ± 0.52 V at 20% RH and 37.65 ± 0.30 V at 30% RH. It was 34.94 ± 0.32 V, 33.76 ± 0.17 V, and 33.38 ± 0.26 V at 40%, 50%, and 60% RH, respectively, before decreasing to 7.15 ± 0.07 V at 70% RH. These amplitudes corresponded to 92.7–104.5% of the 20% RH value over 20–60% RH and 19.9% at 70% RH. Thus, the output was comparatively stable over 20–60% RH but was strongly attenuated at 70% RH. In the local temperature sweep, the voltage amplitude was 27.71 ± 0.20 V at 0 °C, 41.94 ± 0.22 V at 15 °C, 43.09 ± 0.15 V at 30 °C, and 43.74 ± 0.22 V at 45 °C. The output therefore remained similar over 15–45 °C but decreased at 0 °C.

### 4.3. Gesture Recognition Performance

The garment system correctly recognized 1140 of 1200 trials, producing 95.0% overall accuracy. Participant-level accuracy was 95.0 ± 2.3%, ranged from 91% to 98%, and exceeded 90% for every participant. These results show that the three rule-defined gesture classes remained distinguishable across participants after the short familiarization period. The participant-level distribution and confusion matrix quantify recognition consistency across individuals. Separately, the controlled peak-amplitude CV analysis characterizes cycle-, device-, and batch-level electrical repeatability under bench excitation.

For tap, the system correctly classified 361 of 384 trials (94.0%). The remaining tap trials were labeled as double-tap 13 times and as swipe 10 times. Tap produced a localized channel response, so its principal distinction from double-tap was the number and timing of peaks within the recognition window. Light touches and contacts near a sensor edge reduced the consistency of this temporal pattern.

For double-tap, the system correctly classified 381 of 405 trials (94.1%). Twelve trials were labeled as tap and 12 as swipe. The equal number of errors to the other two classes indicates that both incomplete two-peak sequences and unintended movement across adjacent regions contributed to errors. Participant feedback also identified double-tap timing as a source of variation.

**Figure 5:**
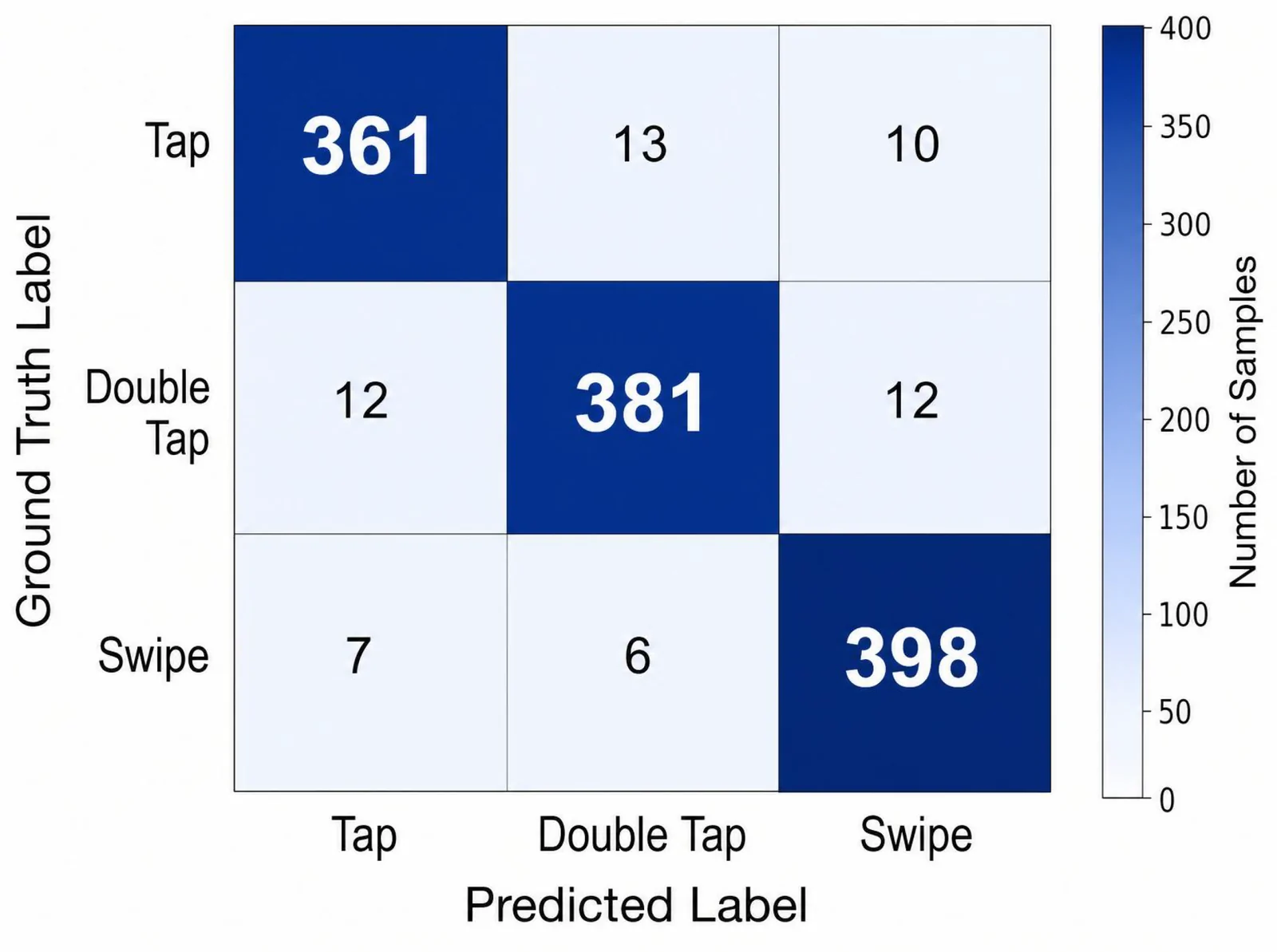
Confusion matrix for tap, double-tap, and swipe recognition across 1200 garment trials from 12 participants.

Swipe produced the highest class accuracy: 398 of 411 trials were recognized correctly (96.8%), with seven labeled as tap and six as double-tap. A swipe activated multiple channels in spatial order, providing a sequence feature in addition to peak count. This channel-order cue made the gesture distinct from the two localized gestures despite variation in movement speed. The confusion matrix in Figure 5 summarizes the three class distributions; participant-level results and the raw response matrix are provided in Supplementary Tables S4 and S5.

### 4.4. Insole Voltage Response and Battery Charging

**Figure 6:**
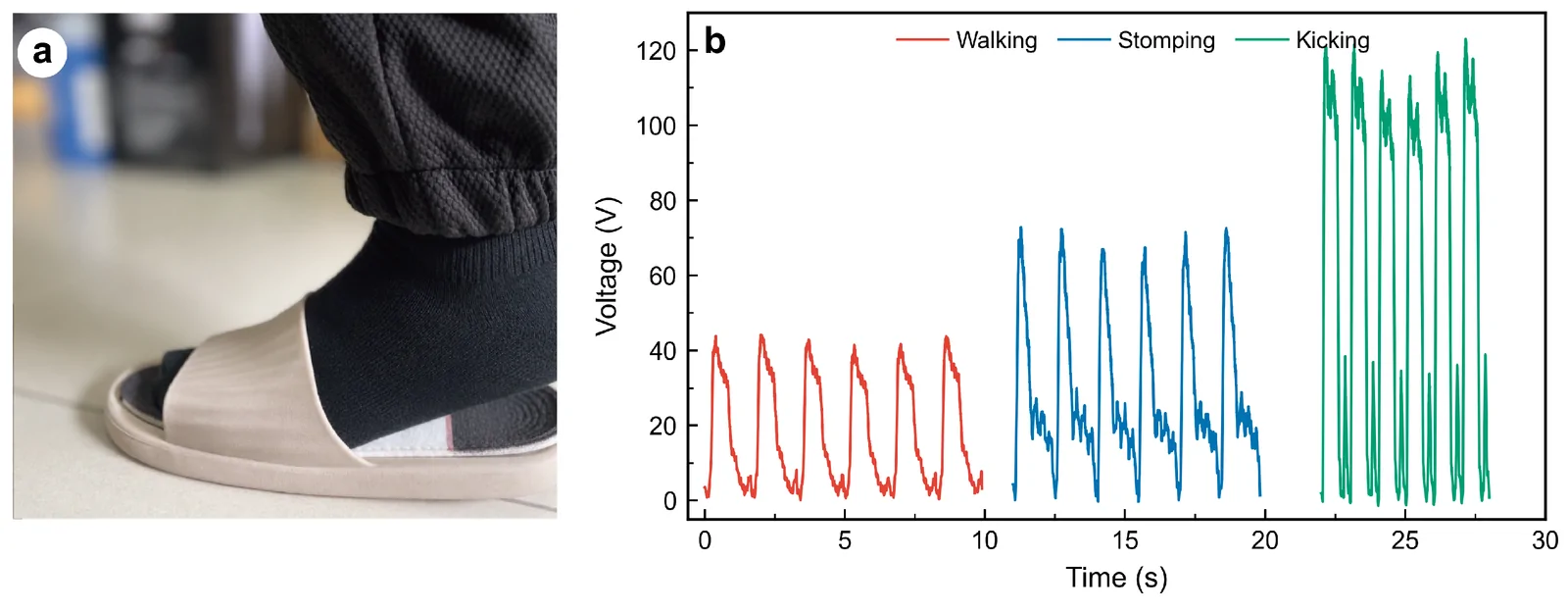
Insole integration and recorded open-circuit voltage. (**a**) TriboTex in the open-toe test slipper. (**b**) Raw 400-Hz traces during walking, stomping, and kicking, with peaks of approximately 44.18 V, 72.83 V, and 123.04 V. The stomping and kicking time coordinates were shifted for visual separation; voltage values were unchanged.

The insole operated in single-electrode mode under repeated vertical contact–separation foot loading. Figure 6b shows the raw 400 Hz voltage traces recorded during walking, stomping, and kicking. The recorded peak open-circuit voltages were approximately 44.18 V, 72.83 V, and 123.04 V, respectively. The three actions produced different peak amplitudes and temporal profiles under natural foot loading. These action-dependent traces document application-level signal differences; a calibrated mechanical comparison would require synchronized force and motion measurements.

In a separate demonstration, the insole output was passed through the rectification and voltage-regulation module shown in Supplementary Figure S6 and connected directly to a battery for charging. This demonstration verifies the implemented rectification, voltage-regulation, and battery-connection path.

### 4.5. Wearability Evaluation

Garment participants rated gesture understandability and system responsiveness at 5.75 ± 0.62 and 5.75 ± 0.45 on the 7-point scale. Controllable input scored 5.50 ± 0.52, overall convenience scored 5.42 ± 0.51, and stability during interaction scored 5.42 ± 0.67. These ratings align with the recognition results and with participants’ descriptions of the interaction. P01 reported that recognition was stable and swipes almost always succeeded on the first try. P04 described the interaction as having a low learning cost that was easy to understand after a short explanation. P08 noted that there were almost no false triggers, making the interaction feel clean. Participants also valued direct mappings between the three gestures and the interface response.

The garment’s material-contact ratings were lower than its interaction ratings. Overall comfort and skin-friendly tactile feel scored 4.50 ± 0.80 and 4.58 ± 1.00. Participants described the coated regions as stiffer and harder than the surrounding cotton, and some compared the surface to a thin plastic layer. Odor and skin sensitivity also affected comfort. Interaction errors were associated with light or edge contact, wet skin, fast swipes, double-tap timing, and displacement of the fabric on the body. These observations connect recognition performance to both contact mechanics and garment attachment.

Several participants provided specific observations about the coating–textile interface. P10 described the surface as somewhat hard, similar to a thin plastic-like fabric, and suggested providing clearer rhythm guidance or improved tolerance for double-tap timing. P07 reported that the prototype felt slightly stuffy after longer contact and that the skin became mildly red, and recommended improving breathability to reduce heat-related skin irritation. P03 noted that the surface felt harder than ordinary cotton fabric and suggested rounding the edges to reduce hard-edge pressure on the hand. These observations indicate that future iterations should address surface hardness, edge transitions, and local breathability in addition to electrical performance.

Regarding interaction errors, participants identified five primary causes: (1) light or tangential finger contact that produced insufficient triboelectric charge (P01, P04, P06); (2) contact near a sensor-region edge that activated adjacent channels (P01, P06); (3) moisture from perspiration associated with less reliable responses (P02, P07, P12); (4) fast swipe speeds that exceeded the 60 Hz sampling window for reliable sequential detection (P03, P08); and (5) inconsistent double-tap timing that fell outside the recognition threshold (P10). P05 reported that errors were more likely when clothing friction caused the fabric to move, and P09 similarly noted that fabric shifting on the body changed the sensor position. These observations suggest that fixation stability and rhythm tolerance are additional priorities for future garment designs. The perspiration-related reports are consistent with the controlled response in Section 4.2: the mean voltage amplitude remained at 33.38–37.65 V over 20–60% RH but decreased to 7.15 V at 70% RH, below the 25- and 30-V decision thresholds. Recognition is therefore likely to become unreliable under such high-humidity conditions. Together, the participant feedback and bench measurements motivate moisture management for reliable on-body operation.

Insole participants rated overall comfort at 4.58 ± 1.24. The highest score was 5.25 ± 0.87 for the absence of heel discomfort or pressure pain. Surface feel, absence of rubbing, and walking stability scored 4.67 ± 0.65, 4.75 ± 0.87, and 4.75 ± 1.29. Participants who matched the available slipper sizes more closely reported cushioning and heel support, while size mismatch produced toe compression, slipping, or uneven arch support. Feedback further identified fixation, thermal comfort, moisture, surface friction, and odor as priorities for footwear integration. Full item wording, means, standard deviations, and participant-level response distributions are provided in Supplementary Tables S6–S10.

Across both prototypes, attachment and fit stability emerged as a shared integration requirement, although the observed consequences differed by mechanical mode. In the garment, fabric shifting changed the position of the patterned regions and was associated with recognition errors (P05, P09). In the insole, size mismatch primarily affected comfort through toe compression, slipping, and uneven pressure; its effect on electrical consistency was not measured. These findings indicate that geometry-flexible patterning must be paired with body-location-specific fixation and fit.

## 5. Discussion

### 5.1. Comparison with Existing Fabrication Methods

Table 2 positions TriboTex alongside other textile-TENG fabrication routes. The comparison focuses on required equipment and explicitly reported time-controlled steps. Because most studies do not report complete end-to-end fabrication time, the literature values are treated as minimum confirmed process times rather than standardized total-time measurements.

TriboTex occupies the masked surface-deposition category. Its central prototyping characteristic is the separation between geometry and deposition: revising the vector stencil retains the material mixture, layer order, deposition settings, pass counts, and curing schedule. The three 20-min drying stages account for 60 min of the less-than-120-min workflow. By comparison, the nylon–Ag–nylon screen-printing process reported by Zhang et al. uses 30 min of drying for each of three sequentially printed layers, corresponding to 90 min of drying before screen preparation, printing, alignment, and handling time are considered [11]. Choi et al. reported 15 min of elastomer mixing followed by 180 min of room-temperature curing, giving at least 195 min before unreported cutting, sewing, and final assembly are included [7]. The literature values represent explicitly reported timed steps rather than standardized end-to-end trials; within this scope, the complete TriboTex workflow has a comparatively short turnaround relative to the timed processing in representative multilayer textile TENGs.

The low consumable cost arises from the use of commercially available nylon solution and Cu-based aerosol coating, an inexpensive replaceable PET stencil, and reusable fabrication equipment, giving a documented core consumable cost of USD 0.003/cm^2^. Manufacturing consistency is supported by retaining the same adhesive stencil throughout all three depositions, which constrains the patterned boundaries and layer registration, and by fixing the spray distance, angle, pass count, and drying conditions. These process controls are consistent with the low within-device, inter-device, and batch-to-batch CVs measured across nine devices from three batches (Supplementary Table S12). Manual alignment and spraying nevertheless remain operator-dependent steps, so the present evidence supports reproducible laboratory prototyping rather than industrial manufacturing tolerances.

Supplementary Table S11 provides a direct comparison with selected fabric-based TENGs using comparatively accessible patterning and coating routes [5,11,44]. The areal power density of TriboTex (1.87 mW/m^2^) was close to the 1.80 mW/m^2^ reported by Jung et al. and lower than the 3.20 and 3.80 mW/m^2^ reported by Zhang et al. and Yi et al., respectively. These values were obtained using different device areas, operating modes, counter-materials, mechanical inputs, current definitions, and power metrics; they are therefore interpreted as contextual benchmarks rather than a controlled performance ranking.

Within this context, the process contribution combines material accessibility, low cost, rapid fabrication and revision, and geometry flexibility to lower the barrier to constructing and revising wearable textile-sensor demos and early-stage experiments.

The electrical results define the operating envelope of this configuration. The voltage and charge increased with force up to 40 N, current scaled with actuation frequency, and the 33-point load sweep located the power-density maximum at 4 GΩ (1870 µW/m^2^, 0.954 µW absolute). The nine-device measurements showed similar force- and frequency-response trends across three fabrication batches. The five-cycle hand-washing result, 8000-cycle contact–separation test, 1000-cycle bending test, and 24-h saline-immersion test together characterize output retention under several conditions relevant to early textile prototyping. The humidity sweep further showed that the voltage amplitude remained at 92.7–104.5% of the 20% RH value over 20–60% RH but decreased to 19.9% at 70% RH. The local temperature sweep showed similar amplitudes from 15 to 45 °C and a reduction to 64.3% of the 30 °C value at 0 °C. These results identify very high humidity and low temperature as conditions that may require environmental calibration or protective integration for unencapsulated use.

The wearable studies provide both functional validation and body-location-specific integration evidence. The eight-channel garment achieved 95.0% gesture accuracy, with participant-level accuracy between 91% and 98%, while the insole produced action-dependent voltage and implemented a rectified, voltage-regulated path connected directly to a battery. Across the two studies, attachment and fit stability emerged as a shared requirement with different observed consequences: garment shifting was associated with recognition errors, whereas insole mismatch affected slipping, pressure distribution, and comfort. Participant reports of perspiration-related errors, considered alongside the controlled humidity response, identify moisture management as a hardware-level priority for reliable sensing and energy capture. Controlled moisture exposure was not performed on the integrated insole, so this requirement remains to be evaluated in the footwear configuration.

### 5.2. Limitations and Future Work

The characterization reported here (nine-device repeatability, four durability conditions, two environmental sweeps, and two 12-participant user studies) establishes the fabrication and functional baseline for TriboTex. The following boundaries define the current scope.

The durability tests covered hand washing, contact–separation cycling, bending fatigue, and simplified saline immersion. Machine laundering, controlled abrasion, adhesion quantification, breathability, and standardized artificial-sweat exposure remain to be measured. The saline-immersion condition (1 wt% NaCl) represents a simplified surrogate rather than a full ISO 105-E04 protocol [45]. The humidity sweep covered 20–70% RH using three isolated textile samples, and the temperature sweep used local cooling/heating at 0–45 °C rather than whole-enclosure conditioning. Combined temperature–humidity cycling was not performed. Garment feedback identified perspiration-related recognition errors that can be interpreted alongside the bench humidity sweep, whereas the integrated insole was not tested under controlled humidity or perspiration. Future footwear evaluation should quantify how in-shoe moisture affects output amplitude, signal consistency, and rectified energy delivery. Both user studies involved 12 participants performing short controlled tasks; extended free-living evaluations were not conducted. The insole traces did not independently control force, contact area, or loading rate, and the battery-charging demonstration verified the rectification path but did not quantify charging rate, stored energy, or efficiency. The two available slipper sizes also coupled insole comfort with footwear fit. Finally, the fabrication comparison is process-based; matched lamination, printing, or weaving baselines were not fabricated under common conditions.

Future work will proceed in three directions:

Extended durability and textile characterization: machine laundering, controlled abrasion, adhesion (ASTM D3359-23) [46], standardized artificial-sweat exposure (ISO 105-E04:2013) [45], combined temperature–humidity cycling, integrated garment and insole humidity testing, and air-permeability measurement.

Expanded wearable evaluation: unconstrained motion studies, larger gesture vocabularies with machine-learning classifiers, size-matched footwear, and synchronized force or inertial sensing for per-step energy quantification and gait-phase segmentation.

Fabrication optimization and benchmarking: full factorial evaluation of laser-cutting, spray-deposition, and curing parameters, together with matched screen-printing, lamination, and embroidery baselines under common geometry, materials, and electrical test conditions.

## 6. Conclusions

TriboTex uses laser-cut adhesive PET stencils and sequential spray deposition to form nylon–Cu–nylon triboelectric regions on cotton textile. The workflow supports geometry changes through the digital stencil and has a documented consumable cost of USD 0.003/cm^2^. Controlled testing produced 52.26 V at 40 N across nine devices from three fabrication batches, a maximum power density of 1870 µW/m^2^ at 4 GΩ, 97.1% voltage retention after five hand-washing cycles, and 99.3% retention across 8000 contact–separation cycles. The output also retained 96.1% of its initial voltage after 1000 bending cycles and 94.2% after 24 h of simplified saline immersion. In the three-sample humidity sweep, voltage amplitude remained at 92.7–104.5% of the 20% RH value over 20–60% RH but decreased to 19.9% at 70% RH. The local temperature sweep showed similar voltage amplitudes from 15 to 45 °C and a lower amplitude at 0 °C. The single-electrode sleeve achieved 95.0% accuracy for three gestures over 1200 trials, and the single-electrode insole generated action-dependent voltage up to 123.04 V and was connected through rectification and voltage regulation to charge a battery. Together, these results demonstrate a reproducible, geometry-flexible textile sensor workflow for rapid application demonstrations and early-stage wearable experiments. Across the two applications, attachment and fit stability emerged as shared integration requirements, while the controlled humidity response and garment feedback identified moisture management as a priority for reliable wearable sensing and energy capture.

## Figures and Tables

**Table 1:** Fabrication parameters for the TriboTex nylon–Cu–nylon stack.

Layer/Step	Material/Equipment	Deposition Setting	Post-Treatment
Stencil preparation	0.225-mm adhesive PET; xTool D1 Pro, 10-W module	100% software power; 8 mm/s; four passes; manual alignment	Adhered to cotton textile
Bottom nylon	Nylon:diluent = 6:4 by weight; 1.0-mm nozzle	Default full output; three passes; approximately 20 cm distance, 80° angle, and 15 s per pass	60 °C for 20 min
Cu-based electrode	Commercial Cu-based conductive aerosol spray	Two passes; approximately 20 cm distance, 80° angle, and 15 s per pass; applied directly from aerosol can	60 °C for 20 min
Top nylon	Nylon:diluent = 6:4 by weight; 1.0-mm nozzle	Default full output; two passes; approximately 20 cm distance, 80° angle, and 15 s per pass	60 °C for 20 min

**Table 2:** Process and reported-time comparison of textile-TENG fabrication routes for wearable prototyping.

Route	Process and Main Equipment	Reported Time Evidence
Stencil-guided spray (TriboTex)	Laser-cut adhesive PET stencil, handheld electric spray gun, and drying oven	Three 20-min layer-drying steps; less than 120 min end-to-end for a standard 3 cm × 3 cm specimen
Layer-by-layer screen printing [11]	Commercial screen printer and 120 °C heating platform; sequential nylon, Ag, and nylon printing	30 min drying for each of three printed layers (90 min drying alone); screen preparation, printing, alignment, and handling time not included
Elastomer coating and textile sewing [7]	Manual Ecoflex/Si-rubber mixing and coating, room-temperature curing, and sewing of the corrugated textile structure	15 min mixing plus 180 min room-temperature curing (at least 195 min); cutting, sewing, and final assembly time not included
Electrospinning [9,35]	High-voltage supply, syringe pump, and collector for forming polymer fibers	At least 1440 min vacuum drying in Bae et al.; electrospinning and final assembly time not included
Dip-coating [5,36,37]	Coating bath and drying equipment for repeated immersion and drying cycles	At least 144 min for the PTFE layer in Yi et al.; CNT processing and device assembly not included
Woven textile electrode [39]	PVC fabric pre-baking, doctor-blade Ag coating, curing, cutting, and weaving	No comparable end-to-end time reported
Embroidery [9]	Computerized embroidery machine and conductive yarn	No comparable end-to-end time reported
Lamination and transfer [10]	Cutting, alignment, and heat-press or adhesive-bonding equipment	No comparable end-to-end time reported

Note: Literature times include only explicitly reported timed steps and should not be interpreted as complete end-to-end fabrication times unless stated otherwise.

## Data Availability

The anonymized participant-level data and study materials supporting the findings of this study are openly available in Zenodo at https://doi.org/10.5281/zenodo.20053213. To protect participant privacy, signed consent forms, completed questionnaire scans, raw photos, raw videos, and other directly identifiable information are not included.

## Supplementary Materials

The following supporting information can be downloaded at https://www.mdpi.com/article/10.3390/s26175554/s1: Figure S1: Four TENG operating modes; Figure S2: Pneumatic contact–separation apparatus; Figure S3: Nylon-layer concentration, thickness, and geometry screening; Figure S4: Garment prototype and recorded eight-channel gesture waveforms; Figure S5: Four-channel garment analog front end; Figure S6: Insole rectification and voltage-regulation module; Figure S7: Representative electrical output waveforms under force and frequency sweeps; Figure S8: Relative resistance change under static bending; Figure S9: Environmental voltage response under relative-humidity and temperature sweeps; Table S1: Stencil, specimen, counter-contact, and laser-cutting details; Table S2: Estimated consumable cost; Table S3: Wearable-prototype electronics and acquisition parameters; Table S4: Garment-study participant characteristics and recognition accuracy; Table S5: Aggregate gesture-recognition counts; Table S6: Garment questionnaire items and summary scores; Table S7: Participant-level garment questionnaire responses; Table S8: Insole-study participant and wearing conditions; Table S9: Insole questionnaire items and summary scores; Table S10: Participant-level insole questionnaire responses; Table S11: Reported electrical performance of selected fabric-based TENGs; Table S12: Within-device, inter-device, and batch-to-batch coefficients of variation; Table S13: Qualitative curing-temperature screening. References cited in the Supplementary Materials are included in the main reference list [5,11,44].
