# Supplementary material for: Low-Cost Spray-Patterned Triboelectric Textiles for Wearable Interaction and Energy Harvesting
Source: Sensors (Basel). 2026 Sep 1;26(17):5554. doi: 10.3390/s26175554 (PMC13568089; doi:10.3390/s26175554)
Supplement: Supplementary file 1 [file sensors-26-05554-s001.zip › sensors-4500855-supplementary.pdf]

sensors

MDPI

Article

Supplementary Materials for "Low-Cost Spray-Patterned Triboelectric Textiles for Wearable Interaction and Energy Harvesting"

Hebo Gong , Shijian Luo * and Ping Shan

College of Computer Science and Technology, Zhejiang University, Hangzhou, Zhejiang, China; gonghebo8@zju.edu.cn (H.G.); shanping@zju.edu.cn (P.S.)
* Correspondence: sjluo@zju.edu.cn

Keywords: triboelectric textile; supplementary materials; fabrication; gesture recognition; wearability

This document provides detailed fabrication dimensions, cost calculations, electronics specifications, additional system figures, participant-level gesture results, a selected literature performance comparison, and full wearability questionnaire data for the TriboTex study.

1. Fabrication and Cost Details

Table S1. Stencil, specimen, counter-contact, and laser-cutting details.

Element	Recorded dimension or setting	Use
Adhesive PET stencil	0.225 mm thickness	Defines spray-patterned nylon and Cu-based regions on cotton
Laser cutter	xTool D1 Pro; 10-W module; 100% software power; 8 mm/s; four passes	Cuts the digital stencil pattern
Standard bench specimen	30 × 30 mm active region on a 40 × 40 mm textile substrate	Force, frequency, load, washing, bending, saline-immersion, and cycling characterization
PTFE counter-contact	0.08 mm thickness; 42 × 17 mm surface; 30 × 17 mm overlap with TriboTex	Contact material for the pneumatic apparatus; effective overlap area 5.10 cm^2
Geometry-study masks	Square, circular, and triangular patterns; nominal areas of 4, 9, 16, and 25 cm^2	Tests the influence of pattern area and shape on V_{OC}

The calculation excludes the cotton substrate, conductive interconnect, electronics, labor, equipment depreciation, energy use, shipping, failed samples, masking waste, gar-

Table S2. Estimated consumable cost of one 100 × 100 mm spray-patterned TriboTex unit under the documented purchase conditions.

Cost item	Calculation basis	Recorded cost
Adhesive PET stencil	Two 100 × 100 mm sheets per patterned unit	RMB 1.20
Nylon dielectric material	≤1.67 g nylon ink per patterned unit	≤RMB 0.13
Nylon-coating diluent	≤1.17 g diluent per patterned unit	≤RMB 0.03
Cu-based conductive coating	≤2.5 mL conductive spray per patterned unit	≤RMB 0.87
**Core consumable subtotal**	Stencil, nylon ink, diluent, and conductive coating	**≤RMB 2.23**
**Area-normalized subtotal**	Subtotal divided by 100 cm^2	**USD 0.003/cm^2**

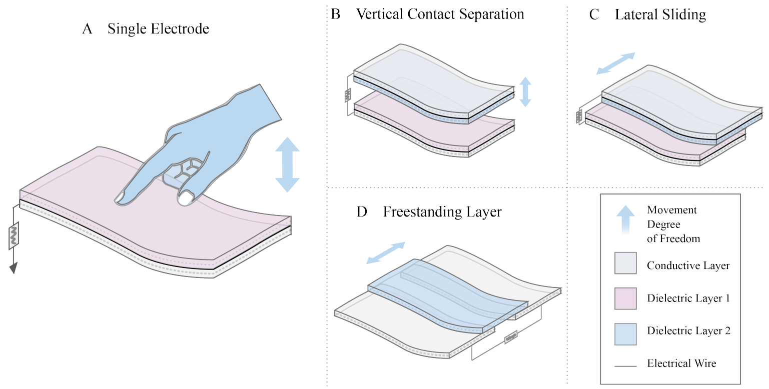

Figure S1. Four TENG operating modes. Both TriboTex wearable prototypes use single-electrode sensing; the insole is mechanically excited by repeated vertical contact–separation loading.

ment or insole carriers, attachment materials, and enclosures. The estimated purchased-component subtotal was approximately RMB 45.00 for the garment signal-recognition module and RMB 10.80 for the insole energy-management module. The garment subtotal included the bare PCB, aggregated electronic components, and one Seeed Studio XIAO; the insole subtotal included its bare PCB and aggregated components.

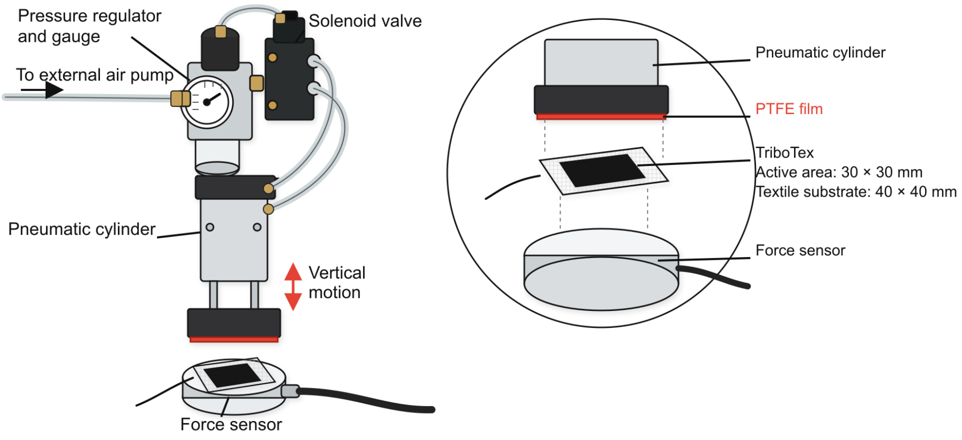

Figure S2. Pneumatic contact–separation apparatus used for controlled electrical characterization. The load cell verified the applied force, and the cylinder provided a nominal 10-mm contact–separation stroke.

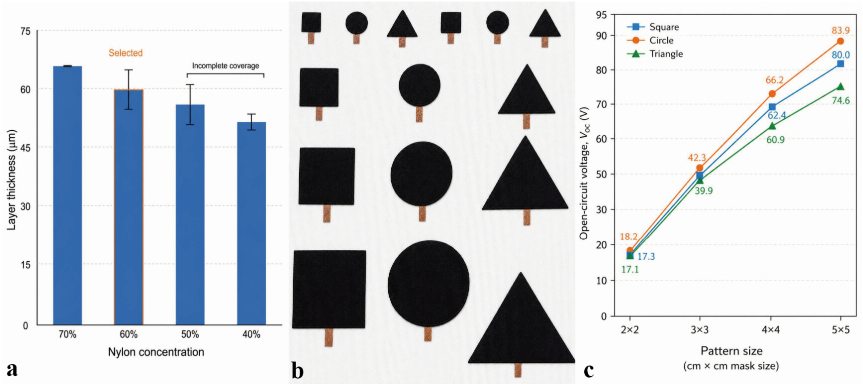

Figure S3. Nylon-layer screening. (a) Average thickness at nylon concentrations of 70%, 60%, 50%, and 40%. The respective averages were approximately 64.3, 59.2, 57.6, and 51.2 μm. Each sample was dried at 60 °C for 15 min, and thickness was measured at five locations. (b) Square, circular, and triangular patterns. (c) V_{OC} for matched nominal pattern areas of 4, 9, 16, and 25 cm^2. At the reported endpoints, the square increased from 17.3 V at 4 cm^2 to 80.0 V at 25 cm^2; the circle reached 83.9 V and the triangle reached 74.6 V at 25 cm^2. Each voltage is the mean of five measurements.

2. Electrical Calculation and Prototype Electronics

For the effective contact area $A = 5.10 \times 10^{-4}$ m^2, current density J was converted to current, power, and area-normalized power density using

$$I = JA, \qquad P = I^2R, \qquad p_A = \frac{P}{A}. \tag{S1}$$

At the measured maximum, $J = 30.2765$ μA m^{-2} and $R = 4$ GΩ, giving $I = 15.44$ nA, $V = IR = 61.76$ V, $P = 0.954$ μW, and $p_A = 1{,}870$ μW m^{-2}.

Static bending produced a progressive increase in normalized resistance. The mean relative changes were 2.68%, 8.04%, and 9.82% at 30°, 60°, and 90°, respectively.

Environmental-response measurements were performed under 20 N and 1 Hz mechanical loading. In the humidity sweep, the voltage amplitude remained between 33.38 and 37.65 V over 20–60% RH before decreasing to 7.15 ± 0.07 V at 70% RH. In the local

Table S3. Wearable-prototype electronics and acquisition parameters.

Subsystem	Component or setting	Recorded value
Garment analog front end	LM324 non-inverting amplifier	$G = 1 + R_f / R_g \approx 1001$
Garment low-pass filter	First-order RC	$R = 10\ \text{k}\Omega$; $C = 1\ \mu\text{F}$; $f_c = (2\pi RC)^{-1} \approx 15.9$ Hz
Garment acquisition	Two four-channel boards and Seeed Studio XIAO	Eight channels; 60 Hz per channel; 480 samples/s aggregate
Garment board size	Customized circular PCB	Approximately 1 cm diameter per four-channel board
Insole battery-charging module	Rectification and voltage-regulation PCB	Approximately 1×2 cm; output connected directly to a battery

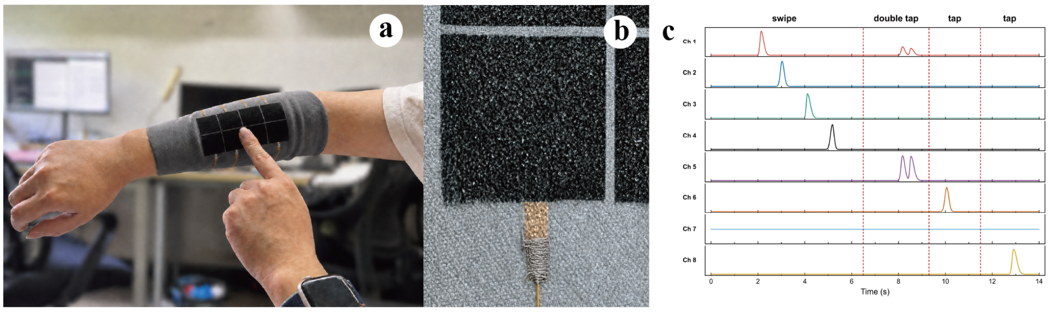

Figure S4. Garment prototype and recorded eight-channel examples. A swipe produced sequential activation across adjacent regions, a double-tap produced two localized peaks, and taps produced single localized transients. The signals were sampled at 60 Hz without smoothing, interpolation, rescaling, normalization, or baseline correction. Region 7 contains the recorded zero-voltage trace, and red dashed lines delimit the annotated gesture intervals.

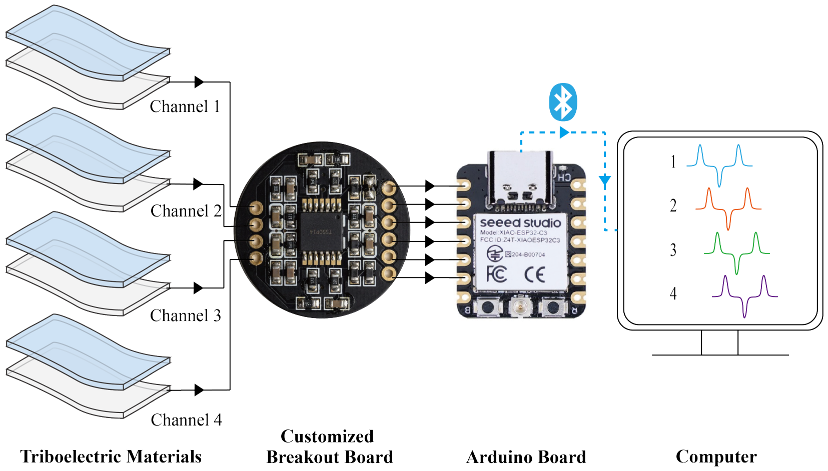

Figure S5. Four-channel garment analog front end. Two boards provided eight conditioned channels to the XIAO and host computer.

temperature sweep, the amplitude increased from 27.71 ± 0.20 V at 0 °C to 41.94 ± 0.22 V at 15 °C and remained between 43.09 and 43.74 V at 30–45 °C.

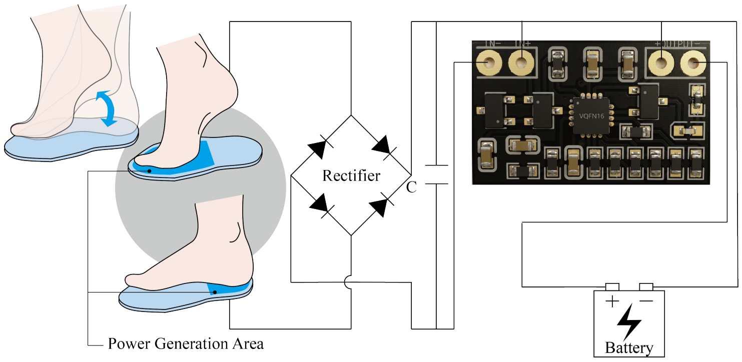

Figure S6. Insole rectification and voltage-regulation module connected directly to a battery for charging.

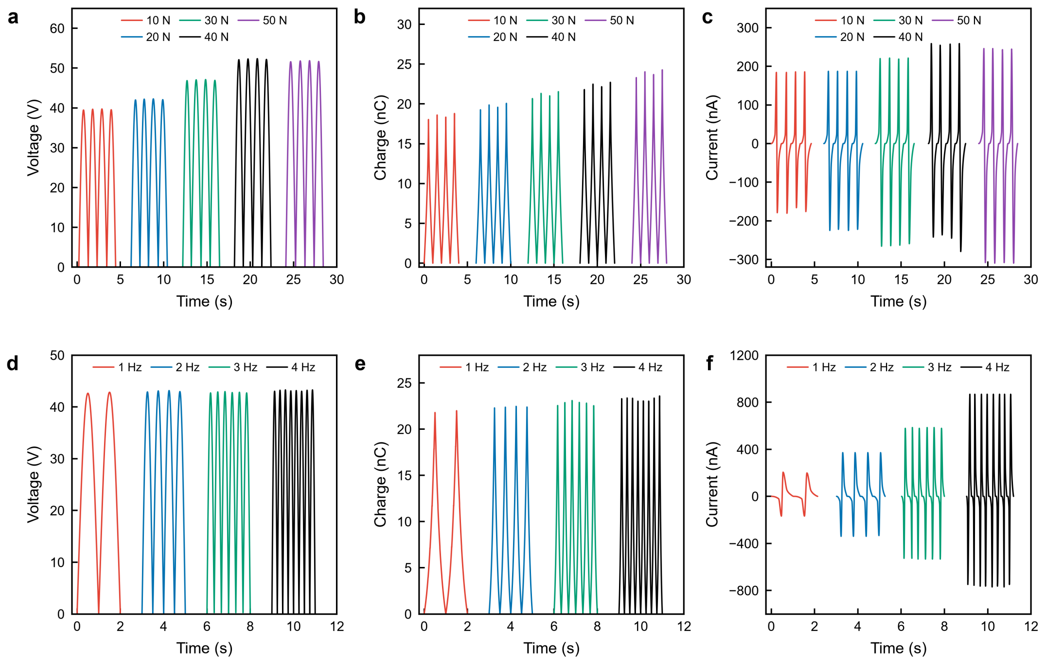

Figure S7. Representative electrical output waveforms under controlled excitation. (a–c) V_{OC}, Q_{SC}, and I_{SC} at 10–50 N and 1 Hz. (d–f) Corresponding waveforms at 1–4 Hz and 20 N. The multi-device peak statistics are reported in Figure 3 of the main manuscript.

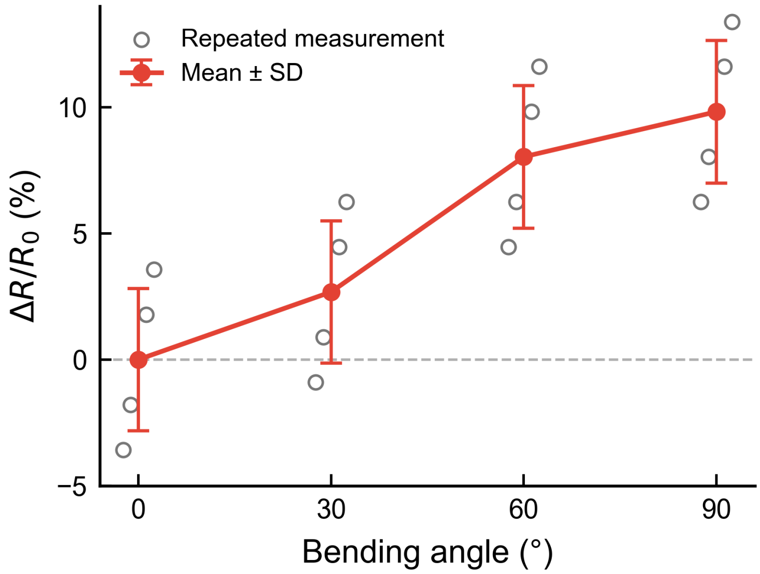

Figure S8. Relative resistance change under static bending. Resistance was normalized to the mean value at 0° using $\Delta R/R_0 = (R - R_0)/R_0$. Gray circles show five repeated measurements at each angle; vermilion points and error bars show the mean ± sample standard deviation of those readings.

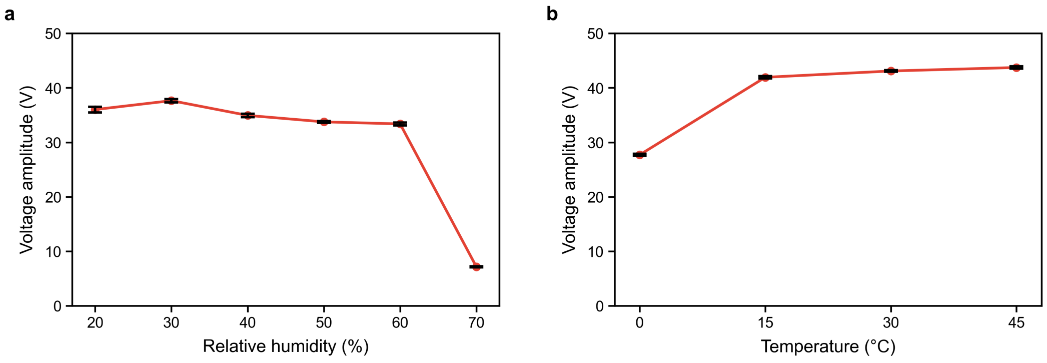

Figure S9. Environmental voltage response under 20 N and 1 Hz mechanical loading. (a) Baseline-corrected voltage amplitude at 20–70% RH in the sealed-enclosure humidity test. (b) Baseline-corrected voltage amplitude at 0, 15, 30, and 45 °C when the tested textile region was locally controlled using a cooling/heating platform. Points and error bars show the mean ± sample standard deviation across three samples ($n = 3$). The temperature test used local thermal exposure rather than whole-enclosure temperature conditioning.

Table S4. Garment-study participant characteristics and participant-level recognition accuracy. Each participant completed 100 trials.

ID	Age	Gender	Dominant hand	Familiarity (1–7)	Skin sensitivity	Accuracy (%)
P01	24	Male	Right	5	No	96
P02	29	Female	Right	6	Yes	93
P03	33	Male	Left	3	No	97
P04	22	Female	Right	4	No	91
P05	27	Male	Right	7	No	97
P06	31	Female	Right	2	No	95
P07	26	Male	Right	4	Yes	92
P08	35	Female	Right	5	No	98
P09	23	Male	Left	3	No	95
P10	28	Female	Right	6	No	96
P11	40	Male	Right	2	No	93
P12	30	Female	Right	5	Yes	97

Table S5. Raw aggregate gesture counts recovered from the archived confusion matrix.

	Predicted label		
**Intended label**	**Tap**	**Double-tap**	**Swipe**
Tap	361	13	10
Double-tap	12	381	12
Swipe	7	6	398

Table S6. Complete garment questionnaire items and summary scores.

Item	Question	Mean ± SD
C1-1	Overall comfort	4.50 ± 0.80
C1-2	Skin-friendly tactile feel	4.58 ± 1.00
C1-3	No discomfort during gestures	5.25 ± 0.62
C2-4	Not easy to slip or shift	5.42 ± 0.67
C2-5	Does not hinder natural movement	5.42 ± 0.51
C2-6	Acceptable for at least 30 min wearing	4.75 ± 0.97
C3-7	Easy to understand gestures	5.75 ± 0.62
C3-8	Controllable input	5.50 ± 0.52
C3-9	Timely system response	5.75 ± 0.45
C3-10	Overall convenience	5.42 ± 0.51
C4-11	Perceived missed recognition	2.08 ± 0.79
C4-12	Perceived false triggering	2.08 ± 0.67

Higher scores indicate more positive ratings for C1–C3. Higher C4 scores indicate stronger perceived recognition errors.

3. Garment Study Data

The row totals are 384 taps, 405 double-taps, and 411 swipes. The class recalls are 94.0%, 94.1%, and 96.8%, respectively, and the total is 1,140 correct predictions among 1,200 trials (95.0%). The participant-level archive contains overall accuracy for each 100-trial block; trial-level predicted labels by participant were not retained.

Table S7. Participant-level garment questionnaire responses on the 7-point scale. Item wording is given in Supplementary Table S6.

ID	C1-1	C1-2	C1-3	C2-4	C2-5	C2-6	C3-7	C3-8	C3-9	C3-10	C4-11	C4-12
P01	5	5	6	6	6	5	6	6	6	6	2	2
P02	4	4	5	5	6	4	6	5	6	5	3	2
P03	5	5	5	6	5	6	5	6	6	5	1	2
P04	4	4	5	5	5	4	6	5	5	5	3	3
P05	5	6	6	6	6	6	7	6	6	6	2	1
P06	5	5	5	5	5	5	5	5	6	5	2	2
P07	3	3	4	5	5	3	6	5	5	5	3	3
P08	6	6	6	6	6	6	6	6	6	6	1	1
P09	4	5	5	4	5	4	5	5	6	5	2	3
P10	5	5	6	5	6	5	6	6	6	6	2	2
P11	4	4	5	6	5	5	5	5	5	5	3	2
P12	4	3	5	6	5	4	6	6	6	6	1	2

4. Insole Study Data

Table S8. Insole-study participant and wearing conditions. All tests lasted 10 min and used open-toe slippers.

ID	Age	Gender	Daily size	Reported condition	Sock type	Slipper size	Activity
P01	22	Female	37	None	Thin	38	Indoor walking
P02	25	Male	42	None	Sports	43	Indoor walking; stairs
P03	31	Female	39	Flat feet or low arches	Thin	38	Indoor walking
P04	28	Male	44	High arches	Sports	43	Indoor walking; stairs
P05	35	Female	38	History of plantar fasciitis or heel pain	Thin	38	Indoor walking
P06	41	Male	41	Previous knee or ankle injury or chronic pain	Thick	43	Indoor walking; stairs
P07	24	Female	36	Prone to blisters or sensitive foot skin	None	38	Indoor walking
P08	30	Male	43	Flat feet or low arches	Thick	43	Indoor walking; running or jumping
P09	47	Female	40	Previous knee, ankle, or lower-back injury or chronic pain	Thin	38	Indoor walking
P10	52	Male	45	None	Sports	43	Indoor walking
P11	20	Female	38	High arches	Thin	38	Indoor walking
P12	33	Male	42	History of plantar fasciitis or heel pain	Sports	43	Indoor walking; stairs

Table S9. Complete insole questionnaire items and summary scores.

Item	Question	Mean ± SD
C1-1	Overall wearing comfort	4.58 ± 1.24
C1-2	Size/shape fits the foot	4.42 ± 1.62
C1-3	Does not noticeably reduce in-shoe space	4.33 ± 1.67
C1-4	Not easy to slip or shift during walking	4.75 ± 1.29
C2-1	No noticeable heel discomfort or pressure pain	5.25 ± 0.87
C2-2	Appropriate arch support	4.58 ± 1.15
C2-3	Forefoot comfort	4.42 ± 1.30
C2-4	Toe movement is not restricted	4.67 ± 1.83
C3-1	Comfortable surface feel	4.67 ± 0.65
C3-2	No obvious rubbing or scratching sensation	4.75 ± 0.87
C3-3	Not noticeably hot	4.42 ± 0.51
C3-4	No obvious sweat/moisture discomfort	4.50 ± 0.67
C4-1	Natural walking without adjusting gait	4.50 ± 1.31
C4-2	No increased fatigue after walking/standing	4.50 ± 1.17

Table S10. Participant-level insole questionnaire responses on the 7-point scale. Item wording is given in Supplementary Table S9.

ID	C1-1	C1-2	C1-3	C1-4	C2-1	C2-2	C2-3	C2-4	C3-1	C3-2	C3-3	C3-4	C4-1	C4-2
P01	6	6	6	6	6	6	6	6	5	6	5	6	6	6
P02	6	6	5	6	6	6	6	6	5	6	5	5	6	6
P03	3	2	2	4	4	3	3	2	4	4	4	4	3	3
P04	4	4	3	5	5	4	3	3	5	5	4	4	4	4
P05	6	6	5	6	6	6	5	6	5	5	5	5	6	6
P06	4	3	6	2	5	4	5	6	5	4	5	5	3	4
P07	4	4	6	3	5	4	5	6	3	3	4	4	4	4
P08	5	5	4	5	6	5	5	5	5	5	4	4	5	5
P09	3	2	2	4	4	3	3	2	4	4	4	4	3	3
P10	3	3	2	5	4	4	2	2	5	5	4	4	3	3
P11	5	6	6	5	6	4	5	6	5	5	5	5	5	5
P12	6	6	5	6	6	6	5	6	5	5	4	4	6	5

5. Selected Literature Performance Comparison

Table S11 compares TriboTex with selected fabric-based TENGs that use comparatively accessible patterning, printing, or coating routes. The entries retain the current type reported by each source rather than converting rectified current, short-circuit current, and short-circuit current density into a nominally common metric.

Table S11. Reported electrical performance of selected fabric-based TENGs using comparatively accessible fabrication routes.

Study	Material, substrate, and fabrication route	Power density (mW/m^2)	Area (cm^2)	Frequency (Hz)	V_{OC} (V)	Reported current
Jung et al. [1]	PU and PI/PDMS and Al patterned on conductive carbon textile	1.80	13.5	1.5	33	0.25 µA (rectified)[a]
**TriboTex (this work)**	Stencil-sprayed nylon/Cu/nylon on cotton; single-electrode configuration with contact–separation against PTFE	**1.87**	5.10	1	42.13	0.202 µA (I_{SC})[b]
Zhang et al. [2]	Screen-printed nylon/Ag/nylon on cotton	3.20	16	1	68	$J_{SC} = 0.056$ µA/cm^2[c]
Yi et al. [3]	Screen-printed Ag electrode with layer-by-layer CNT/cotton and dip-coated PTFE/polyester	3.80	4	1	25	0.060 µA (I_{SC})[d]

[a]Rectified current reported by Jung et al. under the representative 1.5-Hz running condition. [b]Approximate positive short-circuit-current peak for TriboTex at 20 N and 1 Hz; the reported load sweep used the same force and frequency. [c]Zhang et al. reported short-circuit current density rather than total short-circuit current; the source value is retained without conversion. [d]Representative maximum output at 20 N; Yi et al. fixed the frequency at 1 Hz for the power-density/load test. The reported power-density values retain the average, instantaneous, or other definitions used by their sources. Device area, operating mode, counter-material, mechanical excitation, and normalization also differ; therefore, the values should not be interpreted as a controlled ranking.

6. Hierarchical Signal Repeatability Analysis

Table S12 reports coefficients of variation (CVs) for repeated positive peaks. Within-device CV is the mean of nine CVs calculated separately for each device; inter-device CV is calculated from the nine device-level means; and batch-to-batch CV is calculated from three batch means, with three devices in each batch. All CVs use the sample standard deviation. The batch-to-batch values are descriptive because only three fabrication batches were tested.

Table S12. Within-device, inter-device, and batch-to-batch coefficients of variation for positive electrical-output peaks.

Sweep	Signal and condition	Peaks/device	Within-device CV (%)	Inter-device CV (%)	Batch CV (%)
Force, 1 Hz	Positive I_{SC}, 10 N	4	0.25	2.56	1.03
	Positive I_{SC}, 20 N	4	0.25	2.58	0.81
	Positive I_{SC}, 30 N	4	0.21	2.11	0.75
	Positive I_{SC}, 40 N	4	0.18	1.91	1.47
	Positive I_{SC}, 50 N	4	0.19	1.96	1.14
Frequency, 20 N	Positive I_{SC}, 1 Hz	2	0.21	0.31	0.34
	Positive I_{SC}, 2 Hz	4	0.12	0.42	0.47
	Positive I_{SC}, 3 Hz	6	0.07	0.43	0.46
	Positive I_{SC}, 4 Hz	8	0.05	0.39	0.42
Frequency, 20 N	V_{OC}, 1 Hz	2	1.01	1.05	1.17
	V_{OC}, 2 Hz	4	1.08	0.98	1.08
	V_{OC}, 3 Hz	6	1.03	0.98	1.10
	V_{OC}, 4 Hz	8	0.98	1.03	1.13

For the 1-Hz frequency-response recordings, the within-device CV is based on two peaks per device and is therefore descriptive. Inter-device calculations first reduce each device to one mean so that devices receive equal weight. Batch calculations similarly use one mean per fabrication batch.

7. Qualitative Curing-Temperature Screening

Table S13. Qualitative preliminary curing-temperature screening on the cotton textile substrate used to select the standard curing condition.

Temperature	Duration	Qualitative observation	Protocol decision
50 °C	20 min	Coating did not fully dry	Not adopted
60 °C	20 min	Complete drying without visible thermal damage	Adopted
70 °C	15 min	Textile visibly stiffened and developed a harder handle	Not adopted
80 °C	15 min	Visible scorching of the cotton substrate	Not adopted

All trials used the coating applied to cotton textile substrates. Observations were recorded by visual and tactile assessment; no instrumented color, stiffness, or mechanical measurements were performed.

References

1. Jung, S.; Lee, J.; Hyeon, T.; Lee, M.; Kim, D.H. Fabric-Based Integrated Energy Devices for Wearable Activity Monitors. *Advanced Materials* **2014**, *26*, 6329–6334. https://doi.org/10.1002/adma.201402439.
2. Zhang, C.; Zhang, L.; Bao, B.; Ouyang, W.; Chen, W.; Li, Q.; Li, D. Customizing Triboelectric Nanogenerator on Everyday Clothes by Screen-Printing Technology for Biomechanical Energy Harvesting and Human-Interactive Applications. *Advanced Materials Technologies* **2023**, *8*, 2201138. https://doi.org/10.1002/admt.202201138.
3. Yi, J.; Dong, K.; Shen, S.; Jiang, Y.; Peng, X.; Ye, C.; Wang, Z.L. Fully Fabric-Based Triboelectric Nanogenerators as Self-Powered Human-Machine Interactive Keyboards. *Nano-Micro Letters* **2021**, *13*, 103. https://doi.org/10.1007/s40820-021-00621-7.
